# Supplementary material for: Hybrid Models and Biological Model Reduction with PyDSTool
Source: PLoS Comput Biol. 2012 Aug 9;8(8):e1002628. doi: 10.1371/journal.pcbi.1002628 (PMC3415397; doi:10.1371/journal.pcbi.1002628)
Supplement: Text S4 — Complete source code for the PyDSTool package (version 0.88.120504). Includes API documentation and help files linking to web pages. This file is identical to the current public release on Sourceforge.net. (ZIP) [file pcbi.1002628.s004.zip › PyDSTool/html/PyDSTool.Toolbox.optimizers.step.quasi_newton_step-module.html]

xml version="1.0" encoding="ascii"?


PyDSTool.Toolbox.optimizers.step.quasi\_newton\_step


| Home | Trees | Indices | Help | PyDSTool |
| --- | --- | --- | --- | --- |

Package PyDSTool ::
Package Toolbox ::
Package optimizers ::
Package step ::
Module quasi\_newton\_step

[hide private]

[frames] | no frames]

# Module quasi\_newton\_step

source code

Computes a quasi-Newton step for a specific function at a specific
point


|  |  |
| --- | --- |
| Classes | [hide private] |

DFPNewtonStep  
The Davidson-Fletcher-Powell Quasi-Newton step

| Home | Trees | Indices | Help | PyDSTool |
| --- | --- | --- | --- | --- |

|  |  |
| --- | --- |
| Generated by Epydoc 3.0.1 on Fri May 4 15:24:05 2012 | http://epydoc.sourceforge.net |
